# Proximate composition, some phytochemical constituents, potential uses, and safety of neem leaf flour: A review

**DOI:** 10.1002/fsn3.4336

**Published:** 2024-07-17

**Authors:** Kumsa Negasa Andersa, Metekia Tamiru, Tilahun A. Teka, Ibrahim Mohammed Ali, Kasech Tibebu Chane, Tolina Kebede Regasa, Endris Hussen Ahmed

**Affiliations:** ^1^ Department of Post‐Harvest Management, College of Agriculture and Veterinary Medicine Jimma University Jimma Ethiopia; ^2^ Department of Animal Science, College of Agriculture and Veterinary Medicine Jimma University Jimma Ethiopia; ^3^ Department of Plant Science, College of Dryland Agriculture Samara University Samara Ethiopia; ^4^ Department of Agro Food Processing Holeta Polytechnic College, Holeta College Holeta Ethiopia

**Keywords:** food science, neem leaf flour, phytochemical, potential uses, safety

## Abstract

Globally, there is a growing concern about avoiding using artificial compounds in food ingredients, food preservation, and packaging. Among the parts of the neem tree, leaf flour is one of the most commonly used parts in some countries for food and medicinal purposes and is known for containing several nutrients and phytochemicals. In this review, the proximate composition, phytochemical constituents, potential uses, and safety issues of neem leaf flour are discussed. Neem leaf flour contains high levels of crude protein, total carbohydrate, crude fat, and fiber and moderate amounts of crude fat and ash. In addition, it contains numerous health‐promoting phytochemical constituents. Some phytochemicals, such as ascorbic acid, saponin, total alkaloids, carotenoids, total phenols, total flavonoids, and the total antioxidant capacity of neem leaf flour, have been critically discussed. Neem leaf flour has various potential applications in food science, such as preserving foods and preparing food packaging materials. However, researchers' perspectives on its safety are not yet in agreement. In general, the proximate compositions, phytochemical constituents, potential uses, and safety issues of neem leaf flour were compiled and critically reviewed. In addition, research is needed to identify all the toxic substances found in neem leaves and develop methods to eliminate them that hinder their use for various purposes in food. Further research is needed to develop food products from neem leaf flour and evaluate its nutritional value and phytochemical constituents.

## Graphical abstract

Currently, since neem leaf flour is getting great consideration because of its uses for medicinal purposes, preserving grains and fruits, merely using as an ingredient, and as nutraceutical food, it is consumed in the form of tea and paste in some countries.

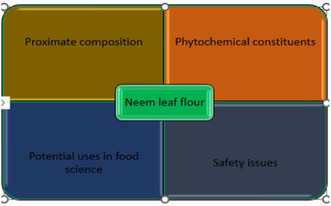

## 1 INTRODUCTION

The use of biopreservatives for preservation, functional food for nutraceutical purposes, and biopolymers for packaging in the food science arena is receiving wide consideration due to their minimal or no impact on consumer health and biodegradability when disposed of after use as packaging materials. Nowadays, the benefits of neem trees for humans have increased worldwide. Neem trees have medicinal and insecticidal properties (Benelli et al., 2017). Almost all parts of the neem serve different purposes in agriculture. Because trees grow in both urban and rural areas, communities can improve their economic conditions by producing products from their seeds and leaves within a short period. In particular, the leaves of neem trees are widely used in various agricultural industries (Vithalkar, 2023).

Neem leaf flour can be consumed in the form of tea and paste, and its tea is used to treat malaria. Neem leaf chutney was a regular part of Mahatma Gandhi's diet, and a nutraceutical tea now being manufactured would have been Gandhi's favorite drink (Kumar et al., 2010). It has also been reported that neem leaf flour is used for food preservation, food packaging preparation, and as an ingredient in some foods (Hosea et al., 2017). The reason for using it for different purposes is its richness in proximate and biologically active compounds. It has been reported that neem leaf flour contains high levels of crude protein and fiber and moderate levels of crude fat and ash (Obikaonu, 2012). It also contains bioactive components with considerable health benefits (Kumar, Mehta, et al., 2018; Kumar, Sharma, et al., 2018). The main bioactive components or active ingredients identified in neem leaf flour are azadirachtin, alkaloids, sodium nimbinate, nimbin, salannin, nimbidin, and quercetin (Alzohairy, 2016). Neem leaf flour is a good source of carotenoids, which are very important in food coloring, and glycoproteins that play vital roles in physiological functions in human life (Goswami et al., 2014; Sarkar et al., 2015).

It has been confirmed that neem leaf flour can prolong the shelf life and quality of tomatoes (Hosea et al., 2017). Other scholars have also concluded that neem derivatives, such as neem leaf flour and oil, can safely preserve grains against pesticides and insecticides (Karthikeyan et al., 2009). Contrary to that, neem leaf flour extract can cause low toxicity and sub‐chronic toxicity in humans, and this can be solved if it is used in a small amount (Braga et al., 2021; Kamatenesi‐Mugisha et al., 2012). One of the most abundant compounds in neem leaves is azadirachtin, which has been confirmed to not affect humans (Islas et al., 2020). However, for the other ingredients, further investigation is needed to determine their toxicity, and establishing a maximum permissible level for each compound in food is essential to ensuring safe usage in food preparation. Although flour contains essential nutrients and active ingredients, its impact on the sensory acceptability of food can be challenging (Kumar et al., 2022). Therefore, due to its safety and pharmacological and nutraceutical properties, it is a potential candidate for biopreservative and therapeutic use (Braga et al., 2021).

**FIGURE 1:**
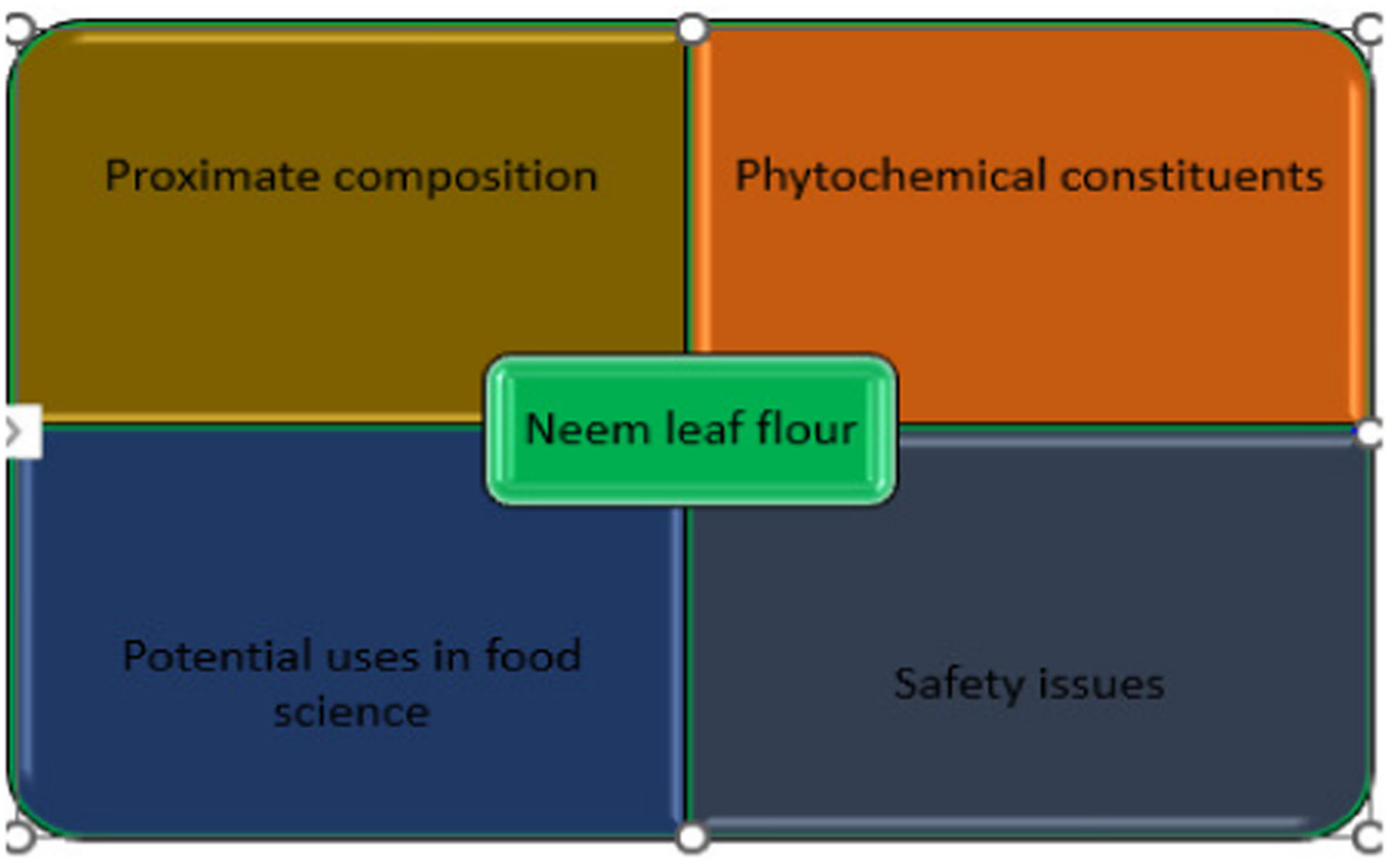
Graphical abstract of the review scope.

Knowledge and information regarding the proximate composition, phytochemical constituents, potential uses, and safety issues of neem leaf flour are crucial for significantly increasing its usage in various food applications, such as food product development, food formulation, and the preservation of perishable agricultural commodities. Therefore, the overall purpose of this review is to provide a comprehensive overview of the proximate composition, phytochemical content, potential uses of neem leaf flour in food science, and safety. A graphic abstract of the scope of the review is shown in Figure 1.

## 2 PROXIMATE COMPOSITION, PHYTOCHEMICAL CONSTITUENTS, POTENTIAL USE OF NEEM LEAF FLOUR IN FOOD, AND SAFETY ISSUES

### 2.1 Proximate composition of neem leaf flour

Neem leaf flour is high in carbohydrate, crude protein, crude fat, and total ash content (Table 1). It has been proven that neem leaf flour is a good source of protein, minerals, and vitamins. Additionally, it contains nutritionally important pigments such as carotenoids (beta‐carotene, alpha‐carotene, and gamma‐carotene), chlorophyll *a*, and chlorophyll *b*. According to the study by Obikaonu (2012), neem leaf flour contains total carbohydrate or nitrogen‐free extract (58.22%), crude protein (18.10%), crude fat (2.50%), crude ash or total mineral (5.26%), and crude fiber (15.56%). On the contrary, another study reported that neem leaf flour is a good source of protein with total carbohydrate (41.90%), crude protein value (22.40%), crude fat (3.0%), crude fiber (20.50), and crude ash (12.20%) (Otache & Agbajor, 2017). A recent study reported that the dry matter, moisture content, crude protein, crude fiber, and crude ash contents of neem leaf flour were 88.94%, 11.06, 20.58%, 14.13, and 11.53%, respectively (Ampode & Asimpen, 2021).

**TABLE 1:** Summary of proximate compositions of neem leaf flour.

Proximate	Min (g/100 g)	References	Max (g/100 g)	References
Moisture content	10.2	Otache and Agbajor (2017)	11.1	Ampode and Asimpen (2021)
Crude protein	7.1	Sharma et al. (2023)	22.4	Otache and Agbajor (2017)
Crude fat	2.5	Obikaonu (2012)	3.2	Otache and Agbajor (2017)
Crude fiber	9.2	Obikaonu (2012)	15.6	Otache and Agbajor (2017)
Total Carbohydrate	22.9	Sharma et al. (2023)	54	Garba and Mungadi (2019)
Crude ash	5.3	Obikaonu (2012)	11.5	Ampode and Asimpen (2021)

Abbreviations: Max, maximum; Min, minimum.

A study conducted by Otache and Agbajor (2017) showed slight variations in the proximate composition of neem leaf flour, reporting that moisture content, protein, fat fiber, and ash were in the range of 14.30%–12.10%, 1.22%–4.04%, 3.18%–2.89%, 10.86%–9.25%, and 4.03%–3.88%, respectively. Another study confirmed that the protein, fat, fiber, and ash contents were 13.42%, 5.17%, 11.93%, and 5.17%, respectively (Atangwho et al., 2009). It was also confirmed that neem leaf flour contains 22.9% and 7.1% carbohydrate and crude protein, respectively (Sharma et al., 2023). However, another study confirmed that the carbohydrate content of the aqueous extract of neem leaves is 54% (Garba & Mungadi, 2019). Neem leaves are also rich in amino acids, such as aspartic, alanine, praline, glutamic, glutamine, cysteine, and other fatty acids. It has been reported that neem leaves are rich in macrominerals such as calcium (0.71%), phosphorus (0.28%), sodium (0.58%), and potassium (2%), and microminerals such as iron (745 ppm), cobalt (10 ppm), manganese (60 ppm), and lead (27 ppm), which are the main constituents of neem leaf flour (Ansari et al., 2012). The proximate compositions of neem leaf flour and its dry matter are indicated below (Table 1).

### 2.2 Some phytochemical constituents of neem leaf flour

Neem leaf flour contains numerous phytochemicals that are chemically diverse and structurally complex. It plays a therapeutic role in managing health due to its various active ingredients (Alzohairy, 2016). The most abundant active constituent in the neem leaf is azadirachtin, and others include alkaloids, saponin, nimbolinin, nimbin, and quercetin. Quercetin and ß‐sitosterol, polyphenolic flavonoids, can be purified from fresh neem leaves and are known to have antibacterial and antifungal properties. Studies conducted on phytochemical analysis of neem leaves have confirmed the presence of high amounts of alkaloids, triterpenes, flavonoids, and saponins, whereas other components, such as catechin and nimbins, appear to be present at lower levels (Tripathi et al., 2016). A previous study has shown that phytochemicals and biopesticidal components are widely available in neem leaf powder or neem leaf flour. The presence of alkaloids, flavonoids, phenolics, saponins, tannins, glycosides, and oxalic acid has been reported. The availability of these compounds could account for the therapeutic uses of neem (Ujah et al., 2021). Some of the main phytochemical constituents of neem leaf flour are indicated below (Table 2).

**TABLE 2:** Summary of phytochemical constituents in neem leaf flour.

Phytochemicals	Min (g/100 g)	References	Max (g/100 g)	References
Ascorbic acid	0.31	Keta et al. (2019)	0.72	Yusuf et al. (2021)
Glycoprotein	33.0	Kundu et al. (2015)	–	–
Saponin	2.4	Aslam et al. (2009)	10	Khanal (2021)
Total alkaloid	4.1	Aslam et al. (2009)	10.7	Khanal (2021)
Carotenoids	0.02	Shrirangasami et al. (2020)	1.10	Hampel et al. (2019)
Total phenolic	0.02	Vergallo et al. (2019)	1.08	Shewale and Rathod (2018)
Total flavonoids	0.52	Vergallo et al. (2019)	13.80	Khanal (2021)
TAC (in DPPH)	71.23	Pokhrel et al. (2015)	80.10	Ahmed et al. (2023)

Abbreviations: DPPH, 1,1 diphenyl‐2‐picrylhydrazyl; Max, maximum; Min, minimum; TAC, total antioxidant capacity.

#### 2.2.1 Ascorbic acid

Ascorbic acid or Vitamin C is a water‐soluble vitamin that is naturally available in some foods. It is a water soluble antioxidant that reacts rapidly with superoxide and peroxyl radicals. Ascorbic acid is an abundantly found vitamin in neem leaves, followed by vitamins E, A, B1, and B2 (Garba & Mungadi, 2019). Another study reported that neem leaves are rich in ascorbic acid and amino acids (Alzohairy, 2016). The quantitative composition of vitamin C in neem leaves is 3154 mg/L or 315.4 mg/100 g (Keta et al., 2019). Ascorbic acid is essential for the formation of collagen, which is necessary for the absorption of iron, some proteins, and folic acid. It prevents the oxidation of other vitamins, facilitates the metabolism of amino acids and calcium, stops internal bleeding, strengthens blood vessels, maintains hard bones and teeth, and heals wounds and burns (Mittu et al., 2022).

#### 2.2.2 Glycoprotein

Glycoproteins are proteins that comprise oligosaccharide chains of carbohydrate and are attached covalently to the amino acid side chains (Yang et al., 2023). They are widely used in the food industry as emulsifiers, stabilizers, and thickening agents, enhancing the shelf life, texture, and stability of products (Liaqat et al., 2023). Glycoproteins play various roles in the food industry, such as combining and stabilizing immiscible substances like oil and water in food products like sauces, dressings, and beverages (Himashree et al., 2022). The leaves of neem trees are rich in glycoproteins named neem leaf glycoprotein (NLGP), which has been shown to have an effective role in restricting tumor growth by modulating local and systematic immunity (Banerjee et al., 2014; Dayakar et al., 2015; Kundu et al., 2015). Recently, it has been proven that neem leaves contain a considerable amount of glycoprotein, which is nontoxic to physiological functions and has a potential role in the treatment of cancer diseases (Bharali et al., 2023).

#### 2.2.3 Saponin

Saponins are glycosidic compounds occurring abundantly in food (Oleszek & Oleszek, 2020). Saponins are naturally occurring compounds that are widely used in food science for their ability to form foam and emulsifying attributes. These properties enable the saponins to be used in beverages such as beers and soft drinks, enhancing the texture and stability of the final product (Schreiner et al., 2021).

Saponins also have potential health benefits, including antioxidant, anticancer, and cholesterol lowering properties. They are being studied for their potential uses as functional ingredients or dietary supplements (Marrelli et al., 2016). Neem leaf contains a considerable content of saponins, which varies depending on several factors such as plant variety, growing conditions, and extraction methods. It has been reported that neem leaves contain 2.4% saponins (Aslam et al., 2009), with variations reported in the range of 2%–10%, depending on agrological factors (Khanal, 2021).

#### 2.2.4 Total alkaloids

Alkaloids are a natural compound that contains nitrogen in its structure and can be found in plants, fungi, and animals (Thawabteh et al., 2019). Alkaloids are known for their use in physiological and pharmacological activities in humans. They are widely available in nature and are found in about 25% of plants. Alkaloids are produced to facilitate the survival of plants in an ecosystem, as they have the potential to act as natural herbicides. Alkaloids are very important as ingredients, supplements, and pharmaceuticals in various applications and medicines in human life. For instance, caffeine in coffee and theobromine in cacao are examples of alkaloids that play a significant role in stimulating the human's brain (Chen & Lin, 2019). Alkaloids are known for their alkaline nature and inhibit a wide array of pharmacological activities. Some alkaloids act as cardiac and respiratory stimulants and are used to treat cancer diseases (Pandey et al., 2014). Phytochemical analysis of methanol neem leaf extracts has shown the presence of alkaloids (Dash et al., 2017). According to the report of Aslam et al. (2009), the amount of alkaloid in neem leaf flour is 4.10 g/100 g, or 4.10% in dry matter. Conversely, a study on quantitative phytochemical analysis has shown that the total alkaloid of neem leaf flour is 10.67 g/100 g, or 10.67% (Khanal, 2021). This difference in the total alkaloid content of neem leaves could be due to variations in the agro‐ecological zones where the trees are grown.

#### 2.2.5 Carotenoids

Carotenoids can be found in yellow, orange, and red‐colored fruits and vegetables (Pezdirc et al., 2016). It has been reported that the carotenoid content of neem leaf flour is 110,000 μg/100 or 1.1% (Hampel et al., 2019). A recent study showed that neem leaf extract has a high carotenoid content (1995 μg/100, or 0.019%) (Shrirangasami et al., 2020).

Carotenoids are among the phytochemical components believed to reduce the risk of developing some degenerative diseases and are responsible for the attractive color of many fruits and vegetables (Sharma et al., 2021). These carotenoids can be used as coloring agents, provitamin A in food and feed, additives in cosmetics, and for preparing multivitamins (Del Campo et al., 2007).

#### 2.2.6 Total phenolic and flavonoid compounds

Phenolic compounds are plant constituents with redox properties responsible for antioxidant activity (Olszowy, 2019). They are found ubiquitously in plants and have potent antioxidant activity mainly due to their redox properties, which allow them to act as reducing agents, hydrogen donors, and chelating agents of metal ions. Various research studies have been conducted on the qualitative and quantitative analysis of the polyphenol compounds in neem leaf powder. The values of total flavonoid content and total phenolic content of neem leaf are reported as 119 mgQE/g and 70 mgGAE/g, respectively (Kumar, Mehta, et al., 2018; Kumar, Sharma, et al., 2018). The total phenolic contents of neem leaf flour dried under optimum conditions were found to be 1080 mg/100 g, or 1.08% (Shewale & Rathod, 2018). Phenolics have gained significant attention due to their health‐promoting properties, combating cancer and neurodegenerative diseases. These health benefits are mainly attributed to its antioxidant properties. Flavonoids are available in higher plants and act as antioxidants, protecting against free radicals that damage cells and tissues. Research has indicated that flavonoids may inhibit the growth of human cancer cells and support the health of the entire cardiovascular system, including arterial walls (Aslam et al., 2009). The content of flavonoids in different extracts of neem leaf flour ranges from 529.5 to 1380 mg/100 g (Vergallo et al., 2019).

#### 2.2.7 Antioxidant capacity

Neem leaves are known for their natural antioxidant properties. An extract of neem leaf flour has shown significant antioxidant activity; therefore, its extract can be used as a natural antioxidant in the preparation of medicines to treat different diseases (Al‐Hashemi & Hossain, 2016). Neem leaves are rich in polyphenol agents with antioxidant properties that can modulate inflammation (Sarkar et al., 2021). The antioxidant activities of neem leaf concentrate have been investigated, and the results have shown that leaf extract or fractions of neem produced in low areas have considerable antioxidant characteristics (Iman et al., 2022). According to the report by Ahmed et al. (2023), the total antioxidant capacity of neem leaves in DPPH was in the range of 69.41% and 80.10%. As indicated in the finding of Pokhrel et al. (2015), the DPPH scavenging activity of neem leaf extract at 50 μg plant extract showed 71.23%, followed by decreasing inhibition activity at lower concentrations. A diet high in antioxidants can reduce the risk of many diseases, such as heart disease and cancer.

Antioxidants scavenge free radicals from body cells and inhibit the damage caused by oxidation (Adwas et al., 2019). Antioxidants are compounds that slow or delay the rate of lipid oxidation in different biological systems (Gupta, 2015). They are a group of chemical substances naturally found in our food that can prevent or minimize oxidative stress in the physiological system (Brar et al., 2013). Antioxidants can be categorized into two types: natural and synthetic. Neem leaf extract has high free radical scavenging activity, with an IC_50_ value of 55.07 μg/mL or 0.06 mg/mL (Abdulkadir et al., 2017). The half maximal inhibitory concentration, or IC_50_ value, is an estimate of a substance's potency in hindering a specific biochemical or biological function. It is a quantity of the substance needed to inhibit, in vitro, a given biological process of a biological component by half (Baltacı et al., 2022).

### 2.3 Potential uses of neem leaf flour

Neem leaves have numerous uses, including the provision of medicine, pesticides, and food. Neem leaf flour has several merits in the food science arena, including roles in both pre‐harvest and post‐harvest management. It is reported that neem leaf flour is used as a natural pesticide and is effective on numerous pests (Debashri & Tamal, 2012). The good amount of proximate composition and phytochemical or biologically active compounds make the flour beneficial to the agricultural community in various ways. Currently, neem leaf flour is extensively used in the agricultural industry worldwide (Vithalkar, 2023). Historically, neem leaf flour has been used as a pesticide and preservative for grains such as maize, beans, wheat, and rice (Boeke et al., 2004; Gajalakshmi & Abbasi, 2004). Neem leaf flour extract has the potential to detoxify aflatoxin B1 and ochratoxin A when applied to wheat, maize, beans, and rice stored for long‐term storage, and it can limit the development of *Aspergillus niger* and *Aspergillus parasiticus* (Mir et al., 2021).

The uses of neem leaf flour are not only restricted to grain preservation but also extend to fruit preservation as nutraceutical food and food packaging materials (Hosea et al., 2017; Kumar et al., 2010; Wylie & Merrell, 2022). It has been confirmed that neem leaf flour has the potential to elongate the shelf life and maintain the quality of tomato fruits during storage (Hosea et al., 2017). It is reported that neem leaf extract is used as a natural pesticide and is effective on numerous pests (Debashri & Tamal, 2012). In southeast Asia, neem leaf flour is consumed in the form of tea and paste, and it is believed that consumption of its tea is used to treat malaria (Kumar et al., 2010). In some countries, communities use neem leaves to prepare food supplements, which are given to consumers in the form of tablets or capsules to address nutritional deficiencies. Its flour extract, in combination with the application of gamma irradiation, can enhance the performance properties of the film that can be used as packaging material (Uthaya Kumar et al., 2020).

Recently, biopolymers made from neem leaf flour extract are gaining attention for a variety of applications in both medicinal and food packaging (Stoleru et al., 2021). In the past couple of years, many groups have demonstrated how neem leaf flour extracts can be incorporated into food preservation films made of environmentally friendly materials like curcumin and turmeric (Wylie & Merrell, 2022). Specifically, neem leaf glycoprotein is nontoxic (Mallick et al., 2013), and there are no residual effects of the aqueous extract of neem leaf on mammals, making neem leaf flour suitable for use as food preservatives (Ali et al., 2021).

Numerous studies have reported that neem leaves are an excellent alternative for feeding poultry as a supplement to improve their feed efficacy, growth, and overall performance, thereby enhancing the availability and quality of food. Additionally, the inclusion of neem leaf flour in animal feed plays a significant role in producing lean, high‐protein meat that helps prevent metabolic and cardiovascular diseases in humans (Singh et al., 2017). The presence of active ingredients or phytochemicals in neem leaf flour is important in preventing the progression of malignant formation, alhzimers, degenerative diseases, and cancer (Hui et al., 2013). The potential uses of neem leaf flour in food science are summarized in Table 3.

**TABLE 3:** Summary of potential uses of neem leaf flour in food science.

Uses of NLF in food science	References
Food preservation	Hosea et al. (2017)
Consumption	Khanal (2021)
Nutraceutical foods	Kumar et al. (2010)
Food packaging material preparation	Wylie and Merrell (2022)
Improve meat quality	Singh et al. (2017)

Abbreviation: NLF, neem leaf flour.

### 2.4 Safety issues of neem leaf flour

Safety is a science‐based discipline, process, or operation to protect foods from the presence of chemicals that could impact human health. In recent years, neem has been recognized as a safe and effective broad‐spectrum antibacterial agent with applications throughout the food industry, from production to consumption and packaging and storage for human consumption (Wylie & Merrell, 2022). However, the available literature regarding its consumption is still not in agreement because of its toxicity. Some researchers have reported intoxication from neem leaf and its derived extract, while others have demonstrated the toxicity of neem leaf flour and its extract in rats, mice, and fish. In industry, the application of neem leaf extract up to 62.5 mg/mL in both vivo and in vitro has increased the survival of shrimp by 76% compared to the untreated group (Morales‐Covarrubias et al., 2016).

It has been reported that the methanolic extract of neem leaves has an LD_50_ of 12 g/kg body weight, whereas the aqueous extract of the leaves showed no toxicity with an LD_50_ of 2 g/kg (Patel et al., 2016). Non‐aqueous extracts in humans have been shown to cause skin allergies (de Oliveira Mesquita et al., 2018; Deng et al., 2013; Patel et al., 2016). Other results revealed that the aqueous extract of neem leaf caused hepatocyte degeneration, leading to animal death due to the presence of nimbolide and nimbic acid in the extract. Later research confirmed the toxicity of neem leaf extract to mice and less toxicity to rats and hamsters (Lisanti et al., 2018). Overall, most studies have focused on the toxicity of the leaf flour extracts, so to make a definitive conclusion regarding the safety of neem leaf flour, conducting further research on the flour is important.

## 3 CONCLUSION

This review evaluated the proximate composition, phytochemical constituents, potential uses of neem leaf flour in food, and safety issues. In this review, all proximate compositions and selected phytochemicals (such as ascorbic acid, carotenoids, total phenolics, total flavonoids, and antioxidant capacity) of the flour were critically evaluated. Neem leaf flour is receiving considerable attention due to its use for medicinal purposes, preserving grains and fruits, as an ingredient in nutraceutical foods, and as a component in tea and paste in certain countries. Additionally, the use of neem leaf flour in food packaging materials is a novel discovery because it can extend the shelf life of foods. This is mainly because it contains important nutrients and phytochemicals and is environmentally friendly compared to other synthetic packaging materials. However, flour contains some compounds that cause sub‐chronic toxicity in mammals, and its sensory attributes may also pose challenges in food products. These issues can be overcome by de‐bittering and masking sensory attributes to increase consumer acceptance and make the product more palatable. Nutrient‐rich and health‐promoting Neem leaf flour should be encouraged and used in food product development and formulation. Therefore, research is needed to develop new food products from neem leaf flour and evaluate its nutritional content to increase its use in various food sectors. Furthermore, further research should focus on identifying the toxic substances found in neem leaves and developing methods to eliminate these substances, which are currently hindering the use of neem leaf flour for various food applications.

## AUTHOR CONTRIBUTIONS

**Kumsa Negasa Andersa:** Conceptualization (equal); data curation (lead); investigation (equal); writing – original draft (lead); writing – review and editing (equal). **Metekia Tamiru:** Conceptualization (equal); data curation (equal); investigation (equal); writing – original draft (equal); writing – review and editing (equal). **Tilahun A. Teka:** Conceptualization (equal); investigation (equal); validation (equal); writing – original draft (equal); writing – review and editing (equal). **Ibrahim Mohammed Ali:** Investigation (equal); writing – original draft (equal); writing – review and editing (equal). **Kasech Tibebu Chane:** Investigation (equal); writing – original draft (equal); writing – review and editing (equal). **Tolina Kebede Regasa:** Investigation (equal); writing – original draft (equal); writing – review and editing (equal). **Endris Hussen Ahmed:** Investigation (equal); writing – original draft (equal); writing – review and editing (equal).

## CONFLICT OF INTEREST STATEMENT

The authors declare no conflicts of interest.

## ETHICS STATEMENT

Ethics approval was not required for this study.

## Data Availability

Additional data will be made available upon request.

## References

[fsn34336-bib-0001] Abdulkadir, A. R. , Mat, N. , & Jahan, M. S. (2017). In‐vitro antioxidant potential in leaf, stem and bark of *Azadirachta indica* . Pertanika Journal of Tropical Agricultural Science, 40(4).

[fsn34336-bib-0002] Adwas, A. A. , Elsayed, A. , Azab, A. E. , & Quwaydir, F. A. (2019). Oxidative stress and antioxidant mechanisms in human body. Journal of Applied Biology & Biotechnology, 6(1), 43–47.

[fsn34336-bib-0003] Ahmed, M. , Marrez, D. A. , Mohamed Abdelmoeen, N. , Abdelmoneem Mahmoud, E. , Ali, M. A. S. , Decsi, K. , & Tóth, Z. (2023). Studying the antioxidant and the antimicrobial activities of leaf successive extracts compared to the green‐chemically synthesized silver nanoparticles and the crude aqueous extract from *Azadirachta indica* . Processes, 11, 1644.

[fsn34336-bib-0004] Al‐Hashemi, Z. S. S. , & Hossain, M. A. (2016). Biological activities of different neem leaf crude extracts used locally in ayurvedic medicine. Pacific Science Review A: Natural Science and Engineering, 18(2), 128–131.

[fsn34336-bib-0005] Ali, E. , Islam, M. S. , Hossen, M. I. , Khatun, M. M. , & Islam, M. A. (2021). Extract of neem (*Azadirachta indica*) leaf exhibits bactericidal effect against multidrug resistant pathogenic bacteria of poultry. Veterinary Medicine and Science, 7(5), 1921–1927. 33955693 10.1002/vms3.511 PMC8464248

[fsn34336-bib-0006] Alzohairy, M. A. (2016). Therapeutics role of *Azadirachta indica* (neem) and their active constituents in diseases prevention and treatment. Evidence‐Based Complementary and Alternative Medicine, 2016, 7382506. 27034694 10.1155/2016/7382506 PMC4791507

[fsn34336-bib-0007] Ampode, K. M. B. , & Asimpen, S. M. (2021). Neem (*Azadirachta indica*) leaf powder as phytogenic feed additives improves the production performance, and immune organ indices of broiler chickens. Journal of Animal Health and Production, 9(4), 362–370.

[fsn34336-bib-0008] Ansari, J. , Khan, S. H. , ul Haq, A. , & Yousaf, M. (2012). Effect of the levels of *Azadirachta indica* dried leaf meal as phytogenic feed additive on the growth performance and haemato‐biochemical parameters in broiler chicks. Journal of Applied Animal Research, 40, 336–345.

[fsn34336-bib-0009] Aslam, F. , Rehman, K. , Asghar, M. , & Sarwar, M. (2009). Antibacterial activity of various phytoconstituents of neem. Pakistan Journal of Agricultural Sciences, 46(3), 209–213.

[fsn34336-bib-0010] Atangwho, I. J. , Ebong, P. E. , Eyong, E. U. , Williams, I. O. , Eten, M. U. , & Egbung, G. E. (2009). Comparative chemical composition of leaves of some antidiabetic medicinal plants: *Azadirachta indica*, *Vernonia amygdalina* and *Gongronema latifolium* . African Journal of Biotechnology, 8(18).

[fsn34336-bib-0077] Baltacı, C. , Öz, M. , Fidan, M. S. , Üçüncü, O. , & Karataş, Ş. M. (2022). Chemical composition, antioxidant and antimicrobial activity of *Colchicum speciosum* Steven growing in Türkiye. Pakistan Journal of Agricultural Sciences, 59(5), 729–736.

[fsn34336-bib-0011] Banerjee, S. , Ghosh, T. , Barik, S. , Das, A. , Ghosh, S. , Bhuniya, A. , Bose, A. , & Baral, R. (2014). Neem leaf glycoprotein prophylaxis transduces immune dependent stop signal for tumor angiogenic switch within tumor microenvironment. PLoS One, 9(11), e110040. 25391149 10.1371/journal.pone.0110040 PMC4229107

[fsn34336-bib-0012] Benelli, G. , Canale, A. , Toniolo, C. , Higuchi, A. , Murugan, K. , Pavela, R. , & Nicoletti, M. (2017). Neem (*Azadirachta indica*): Towards the ideal insecticide? Natural Product Research, 31(4), 369–386. 27687478 10.1080/14786419.2016.1214834

[fsn34336-bib-0013] Bharali, P. J. , Bordoloi, S. K. , Swarnamoni, D. A. S. , & Lahon, K. (2023). Effect of chronic administration of aqueous extract of neem (*Azadirachta indica*) leaves on paracetamol‐induced hepatotoxicity in Wistar albino rats. Current Perspectives on Medicinal and Aromatic Plants, 5(2), 146–161.

[fsn34336-bib-0014] Boeke, S. J. , Boersma, M. G. , Alink, G. M. , van Loon, J. J. , van Huis, A. , Dicke, M. , & Rietjens, I. M. (2004). Safety evaluation of neem (*Azadirachta indica*) derived pesticides. Journal of Ethnopharmacology, 94(1), 25–41. 15261960 10.1016/j.jep.2004.05.011

[fsn34336-bib-0015] Braga, T. M. , Rocha, L. , Chung, T. Y. , Oliveira, R. F. , Pinho, C. , Oliveira, A. I. , Morgado, J. , & Cruz, A. (2021). *Azadirachta indica* A. Juss. In vivo toxicity—An updated review. Molecules, 26(2), 252. 33419112 10.3390/molecules26020252 PMC7825405

[fsn34336-bib-0016] Brar, S. K. , Dhillon, G. S. , & Soccol, C. R. (Eds). (2013). Biotransformation of waste biomass into high value biochemicals. Springer Science & Business Media.

[fsn34336-bib-0017] Chen, C. , & Lin, L. (2019). Alkaloids in diet. In Handbook of dietary phytochemicals (pp. 1–35).

[fsn34336-bib-0018] Dash, S. P. , Dixit, S. , & Sahoo, S. (2017). Phytochemical and biochemical characterizations from leaf extracts from *Azadirachta indica*: An important medicinal plant. Biochemistry and Analytical Biochemistry, 6(323), 1–4.

[fsn34336-bib-0019] Dayakar, A. , Chandrasekaran, S. , Veronica, J. , Sundar, S. , & Maurya, R. (2015). In vitro and in vivo evaluation of anti‐leishmanial and immunomodulatory activity of neem leaf extract in *Leishmania donovani* infection. Experimental Parasitology, 153, 45–54. 25747203 10.1016/j.exppara.2015.02.011

[fsn34336-bib-0078] de Oliveira Mesquita, F. , Oliveira Batista, R. , Cavalcante, L. F. , Bezerra Costa, F. G. , de Luna Souto, A. G. , da Costa Leite Coelho, D. , da Silva, K. B. , & de Oliveira Filho, F. X. (2018). Behavior of neem seedlings (*'Azadirachta indica*') irrigated with saline water in the soil with biofertilizer and drainage. Australian Journal of Crop Science, 12(12), 1950–1956.

[fsn34336-bib-0020] Debashri, M. , & Tamal, M. (2012). A review on efficacy of *Azadirachta indica* A. Juss based biopesticides: An Indian perspective. Research Journal of Recent Sciences, 1, 94–99.

[fsn34336-bib-0021] Del Campo, J. A. , García‐González, M. , & Guerrero, M. G. (2007). Outdoor cultivation of microalgae for carotenoid production: Current state and perspectives. Applied Microbiology and Biotechnology, 74, 1163–1174. 17277962 10.1007/s00253-007-0844-9

[fsn34336-bib-0022] Deng, Y. X. , Cao, M. , Shi, D. X. , Yin, Z. Q. , Jia, R. Y. , Xu, J. , Wang, C. , Lv, C. , Liang, X. X. , He, C. L. , & Yang, Z. R. (2013). Toxicological evaluation of neem (*Azadirachta indica*) oil: Acute and subacute toxicity. Environmental Toxicology and Pharmacology, 35(2), 240–246. 23353547 10.1016/j.etap.2012.12.015

[fsn34336-bib-0023] Gajalakshmi, S. , & Abbasi, S. A. (2004). Neem leaves as a source of fertilizer‐cum‐pesticide vermicompost. Bioresource Technology, 92(3), 291–296. 14766163 10.1016/j.biortech.2003.09.012

[fsn34336-bib-0024] Garba, S. , & Mungadi, H. U. (2019). Quantitative chemical compositions of neem (*Azadirachta indica*) leaf aqueous extracts in Sokoto, Nigeria. International Journal of Research and Scientific Innovation, 6(7), 2–321.

[fsn34336-bib-0025] Goswami, K. K. , Barik, S. , Sarkar, M. , Bhowmick, A. , Biswas, J. , Bose, A. , & Baral, R. (2014). Targeting STAT3 phosphorylation by neem leaf glycoprotein prevents immune evasion exerted by supraglottic laryngeal tumor induced M2 macrophages. Molecular Immunology, 59(2), 119–127. 24607970 10.1016/j.molimm.2014.01.015

[fsn34336-bib-0026] Gupta, D. (2015). Methods for determination of antioxidant capacity: A review. International Journal of Pharmaceutical Sciences and Research, 6(2), 546.

[fsn34336-bib-0027] Hampel, H. , Vergallo, A. , Perry, G. , Lista, S. , & Alzheimer Precision Medicine Initiative . (2019). The Alzheimer precision medicine initiative. Journal of Alzheimer's Disease, 68(1), 1–24. 10.3233/JAD-181121 30814352

[fsn34336-bib-0028] Himashree, P. , Sengar, A. S. , & Sunil, C. K. (2022). Food thickening agents: Sources, chemistry, properties and applications – A review. International Journal of Gastronomy and Food Science, 27, 100468.

[fsn34336-bib-0029] Hosea, Z. Y. , Liamngee, K. , Owoicho, A. L. , & David, T. (2017). Effect of neem leaf powder on post harvest shelf life and quality of tomato fruits in storage. International Journal of Development and Sustainability, 6(10), 1334–1349.

[fsn34336-bib-0030] Hui, C. , Qi, X. , Qianyong, Z. , Xiaoli, P. , Jundong, Z. , & Mantian, M. (2013). Flavonoids, flavonoid subclasses and breast cancer risk: A meta‐analysis of epidemiologic studies. PLoS One, 8(1), e54318. 23349849 10.1371/journal.pone.0054318 PMC3548848

[fsn34336-bib-0031] Iman, M. , Taheri, M. , & Bahari, Z. (2022). The anti‐cancer properties of neem (*Azadirachta indica*) through its antioxidant activity in the liver: Its pharmaceutics and toxic dosage forms. A literature review. Journal of Complementary and Integrative Medicine, 19(2), 203–211. 33964199 10.1515/jcim-2021-0009

[fsn34336-bib-0032] Islas, J. F. , Acosta, E. , Zuca, G. , Delgado‐Gallegos, J. L. , Moreno‐Treviño, M. G. , Escalante, B. , & Moreno‐Cuevas, J. E. (2020). An overview of neem (*Azadirachta indica*) and its potential impact on health. Journal of Functional Foods, 74, 104171.

[fsn34336-bib-0033] Kamatenesi‐Mugisha, M. , Buyungo, J. P. , Egwang, P. , Vudriko, P. , Gakunga, J. N. , Deng, A. , Ogendo, J. , & Mihale, J.M. (2012). Evaluation of the biosafety of selected botanical pesticide plants used by subsistence farmers around the Lake Victoria basin (pp. 45–57).

[fsn34336-bib-0034] Karthikeyan, C. , Veeraragavathatham, D. , Karpagam, D. , & Firdouse, S.A. (2009). Traditional storage practices.

[fsn34336-bib-0035] Keta, J. N. , Suberu, H. A. , Shehu, K. , Yahayya, U. , Mohammad, N. K. , & Gudu, G. B. (2019). Effect of neem (*Azadirachta indica* A. Juss) leaf extract on the growth of aspergillus species isolated from foliar diseases of rice (*Oryzea sativa*). Science World Journal, 14(1), 98–102.

[fsn34336-bib-0036] Khanal, S. (2021). Qualitative and quantitative phytochemical screening of *Azadirachta indica* Juss. Plant parts. International Journal of Applied Sciences and Biotechnology, 9(2), 122–127.

[fsn34336-bib-0037] Kumar, K. P. , Bhowmik, D. , Tripathi, K. K. , & Chandira, M. (2010). Traditional Indian herbal plants tulsi and its medicinal importance. Research Journal of Pharmacognosy and Phytochemistry, 2(2), 93–101.

[fsn34336-bib-0038] Kumar, R. , Mehta, S. , & Pathak, S. R. (2018). Bioactive constituents of neem. In Synthesis of medicinal agents from plants (pp. 75–103). Elsevier.

[fsn34336-bib-0039] Kumar, R. , Sharma, S. , & Devi, L. (2018). Investigation of total phenolic, flavonoid contents and kundu khanalantioxidant activity from extracts of *Azadirachta indica* of Bundelkhand region. International Journal of Life Sciences Research, 2455(1716), 1716.

[fsn34336-bib-0040] Kumar, S. , Singh, N. , Devi, L. S. , Kumar, S. , Kamle, M. , Kumar, P. , & Mukherjee, A. (2022). Neem oil and its nanoemulsion in sustainable food preservation and packaging: Current status and future prospects. Journal of Agriculture and Food Research, 7, 100254.

[fsn34336-bib-0041] Kundu, P. I. , Barik, S. U. , Sarkar, K. O. , Bose, A. N. , Baral, R. A. , & Laskar, S. U. (2015). Chemical investigation of neem leaf glycoprotein used as immunoprophylactic agents for tumor growth restriction. International Journal of Pharmacy and Pharmaceutical Sciences, 7, 195–199.

[fsn34336-bib-0043] Liaqat, A. , Chughtai, M. F. J. , Khaliq, A. , Farooq, U. , Shahbaz, M. , Ali, A. , Saeed, K. , Sameed, N. , Kanwal, M. , Wattoo, A. G. , & Iqbal, R. (2023). Applications of biosurfactants in dairy industry. In Applications of next generation biosurfactants in the food sector (pp. 509–526). Academic Press.

[fsn34336-bib-0044] Lisanti, E. , Sajuthi, D. , Agil, M. , Arifiantini, R. , & Winarto, A. (2018). The effect of aqueous seed extract of neem (*Azadirachta indica* A. Juss) on liver histology of male mice (*Mus musculus albinus*). In AIP conference proceedings (Vol. 2019, No. 1). AIP Publishing.

[fsn34336-bib-0045] Mallick, A. , Ghosh, S. , Banerjee, S. , Majumder, S. , Das, A. , Mondal, B. , Barik, S. , Goswami, K. K. , Pal, S. , Laskar, S. , & Sarkar, K. (2013). Neem leaf glycoprotein is nontoxic to physiological functions of Swiss mice and Sprague Dawley rats: Histological, biochemical and immunological perspectives. International Immunopharmacology, 15(1), 73–83. 23178577 10.1016/j.intimp.2012.11.006

[fsn34336-bib-0046] Marrelli, M. , Conforti, F. , Araniti, F. , & Statti, G. A. (2016). Effects of saponins on lipid metabolism: A review of potential health benefits in the treatment of obesity. Molecules, 21(10), 1404. 27775618 10.3390/molecules21101404 PMC6273086

[fsn34336-bib-0047] Mir, S. A. , Dar, B. N. , Shah, M. A. , Sofi, S. A. , Hamdani, A. M. , Oliveira, C. A. , Moosavi, M. H. , Khaneghah, A. M. , & Sant'Ana, A. S. (2021). Application of new technologies in decontamination of mycotoxins in cereal grains: Challenges, and perspectives. Food and Chemical Toxicology, 148, 111976. 33422602 10.1016/j.fct.2021.111976

[fsn34336-bib-0048] Mittu, B. , Bhat, Z. R. , Chauhan, A. , Kour, J. , Behera, A. , & Kaur, M. (2022). Ascorbic acid. In Nutraceuticals and health care (pp. 289–302). Academic Press.

[fsn34336-bib-0049] Morales‐Covarrubias, M. S. , García‐Aguilar, N. , del Carmen Bolan‐Mejía, M. , & Puello‐Cruz, A. C. (2016). Evaluation of medicinal plants and colloidal silver efficiency against *Vibrio parahaemolyticus* infection in *Litopenaeus vannamei* cultured at low salinity. Diseases of Aquatic Organisms, 122(1), 57–65. 27901504 10.3354/dao03060

[fsn34336-bib-0050] Obikaonu, H. O. (2012). Evaluation of the nutritional value of neem (*Azadirachta indica*) leaf meal on the performance of finisher broilers. International Journal of Agriculture and Rural Development, 15(3), 1235–1239.

[fsn34336-bib-0051] Oleszek, M. , & Oleszek, W. (2020). Saponins in food. In Handbook of dietary phytochemicals (pp. 1–40).

[fsn34336-bib-0052] Olszowy, M. (2019). What is responsible for antioxidant properties of polyphenolic compounds from plants? Plant Physiology and Biochemistry, 144, 135–143. 31563754 10.1016/j.plaphy.2019.09.039

[fsn34336-bib-0053] Otache, M. A. , & Agbajor, G. K. (2017). Proximate and mineral composition of leaves of *Azadirachta indica* . International Journal of Current Research in Chemistry and Pharmaceutical Sciences, 4(11), 50–54.

[fsn34336-bib-0054] Pandey, G. , Verma, K. K. , & Singh, M. (2014). Evaluation of phytochemical, antibacterial and free radical scavenging properties of *Azadirachta indica* (neem) leaves. International Journal of Pharmacy and Pharmaceutical Sciences, 6(2), 444–447.

[fsn34336-bib-0055] Patel, S. M. , Venkata, K. C. N. , Bhattacharyya, P. , Sethi, G. , & Bishayee, A. (2016). Potential of neem (*Azadirachta indica* L.) for prevention and treatment of oncologic diseases. Seminars in Cancer Biology, 40–41, 100–115. 10.1016/j.semcancer.2016.03.002 27019417

[fsn34336-bib-0056] Pezdirc, K. , Hutchesson, M. J. , Williams, R. L. , Rollo, M. E. , Burrows, T. L. , Wood, L. G. , Oldmeadow, C. , & Collins, C. E. (2016). Consuming high‐carotenoid fruit and vegetables influences skin yellowness and plasma carotenoids in young women: A single‐blind randomized crossover trial. Journal of the Academy of Nutrition and Dietetics, 116(8), 1257–1265. 27160341 10.1016/j.jand.2016.03.012

[fsn34336-bib-0057] Pokhrel, B. , Rijal, S. , Raut, S. , & Pandeya, A. (2015). Investigations of antioxidant and antibacterial activity of leaf extracts of *Azadirachta indica* . African Journal of Biotechnology, 14(46), 3159–3163.

[fsn34336-bib-0058] Sarkar, P. , Dh, L. K. , Dhumal, C. , Panigrahi, S. S. , & Choudhary, R. (2015). Traditional and ayurvedic foods of Indian origin. Journal of Ethnic Foods, 2(3), 97–109.

[fsn34336-bib-0076] Sarkar, S. , Singh, R. P. , & Bhattacharya, G. (2021). Exploring the role of *Azadirachta indica* (neem) and its active compounds in the regulation of biological pathways: An update on molecular approach. 3 Biotech, 11(4), 178. 10.1007/s13205-021-02745-4 PMC7981372 33927969

[fsn34336-bib-0059] Schreiner, T. B. , Colucci, G. , Santamaria‐Echart, A. , Fernandes, I. P. , Dias, M. M. , Pinho, S. P. , & Barreiro, M. F. (2021). Evaluation of saponin‐rich extracts as natural alternative emulsifiers: A comparative study with pure Quillaja Bark saponin. Colloids and Surfaces A: Physicochemical and Engineering Aspects, 623, 126748.

[fsn34336-bib-0060] Sharma, N. , Singh, K. , Tripathi, C. C. , & Bera, M. K. (2023). Resistive switching in neem (*Azadirachta indica*) thin film for a cost‐effective and washable biomemristor. Journal of Materials Science: Materials in Electronics, 34(1), 50.

[fsn34336-bib-0075] Sharma, S. , Katoch, V. , Kumar, S. , & Chatterjee, S. (2021). Functional relationship of vegetable colors and bioactive compounds: Implications in human health. The Journal of Nutritional Biochemistry, 92, 108615. 33705954 10.1016/j.jnutbio.2021.108615

[fsn34336-bib-0061] Shewale, S. , & Rathod, V. K. (2018). Extraction of total phenolic content from *Azadirachta indica* or (neem) leaves: Kinetics study. Preparative Biochemistry and Biotechnology, 48(4), 312–320. 29424626 10.1080/10826068.2018.1431784

[fsn34336-bib-0063] Shrirangasami, S. R. , Murugaragavan, R. , Rakesh, S. S. , & Ramesh, P. T. (2020). Chemistry behind in neem (*Azadirachta indica*) as medicinal value to living forms – A review. Journal of Pharmacognosy and Phytochemistry, 9(6), 467–469.

[fsn34336-bib-0064] Singh, M. K. , Sharma, R. K. , & Singh, S. K. (2017). Neem leaf meal supplementation for profitable poultry production: A review. Indian Journal of Poultry Science, 52(3), 239.

[fsn34336-bib-0065] Stoleru, E. , Irimia, A. , & Butnaru, E. (2021). Bio‐based bioplastics in active food packaging. Bioplastics for Sustainable Development, 347–379.

[fsn34336-bib-0066] Thawabteh, A. , Juma, S. , Bader, M. , Karaman, D. , Scrano, L. , Bufo, S. A. , & Karaman, R. (2019). The biological activity of natural alkaloids against herbivores, cancerous cells and pathogens. Toxins, 11(11), 656. 31717922 10.3390/toxins11110656 PMC6891610

[fsn34336-bib-0067] Tripathi, S. , Jadaun, J. S. , Chandra, M. , & Sangwan, N. S. (2016). Medicinal plant transcriptomes: The new gateways for accelerated understanding of plant secondary metabolism. Plant Genetic Resources, 14(4), 256–269.

[fsn34336-bib-0068] Ujah, I. I. , Nsude, C. A. , Ani, O. N. , Alozieuwa, U. B. , Okpako, I. O. , & Okwor, A. E. (2021). Phytochemicals of neem plant (*Azadirachta indica*) explains its use in traditional medicine and pest control. GSC Biological and Pharmaceutical Sciences, 14(2), 165–171.

[fsn34336-bib-0069] Uthaya Kumar, U. S. , Abdulmadjid, S. N. , Olaiya, N. G. , Amirul, A. A. , Rizal, S. , Rahman, A. A. , Alfatah, T. , Mistar, E. M. , & Abdul Khalil, H. P. S. (2020). Extracted compounds from neem leaves as antimicrobial agent on the physico‐chemical properties of seaweed‐based biopolymer films. Polymers, 12(5), 1119. 32422913 10.3390/polym12051119 PMC7284887

[fsn34336-bib-0070] Vergallo, C. , Panzarini, E. , & Dini, L. (2019). High performance liquid chromatographic profiling of antioxidant and antidiabetic flavonoids purified from *Azadirachta indica* (neem) leaf ethanolic extract. Pure and Applied Chemistry, 91(10), 1631–1640.

[fsn34336-bib-0071] Vithalkar, A. (2023). The green gold‐neem: A review. Tijer‐International Research Journal, 10(1), 62–77.

[fsn34336-bib-0072] Wylie, M. R. , & Merrell, D. S. (2022). The antimicrobial potential of the neem tree *Azadirachta indica* . Frontiers in Pharmacology, 13, 891535. 35712721 10.3389/fphar.2022.891535 PMC9195866

[fsn34336-bib-0073] Yang, Z. , Sarkar, A. K. , & Amdursky, N. (2023). Glycoproteins as a platform for making proton‐conductive free‐standing biopolymers. Biomacromolecules, 24(3), 1111–1120. 36787188 10.1021/acs.biomac.2c01007

[fsn34336-bib-0074] Yusuf, R. , Fuadi, I. , Istina, I. N. , Swastika, S. , & Fahri, A. (2021). Extract of fermentated plants to increased content of vitamin c, total phenol and antioxidant of mustard greens. In IOP conference series: Earth and environmental science (Vol. 803, No. 1, p. 012022). IOP Publishing.

